# Pregnant Women’s Experiences with Midwifery-Led Antenatal Care Services in Peri-Urban Communities in Karachi, Pakistan

**DOI:** 10.2147/PROM.S404476

**Published:** 2023-05-09

**Authors:** Nida Salman Yazdani, Kaniz Amna Haider, Amna Khan, Syed Ali Jaffar Zaidi, Akbar Rajani, Imran Nisar, Fyezah Jehan, Zahra Hoodbhoy

**Affiliations:** 1 Research and Programs, VITAL Pakistan Trust, Karachi, Pakistan; 2 Department of Paediatrics and Child Health, the Aga Khan University, Karachi, Pakistan

**Keywords:** antenatal care, patient satisfaction, Respectful Maternity Care, Pakistan

## Abstract

**Purpose:**

To understand pregnant women’s experience with midwifery-led antenatal care services using the Respectful Maternity Care charter in primary health centers in Karachi, Pakistan.

**Methods:**

This cross-sectional study was at Rehri Goth and Ibrahim Hyderi, two peri-urban communities in Karachi, Pakistan, where women receive antenatal care services. All pregnant women in their third trimester who consented during the study period were included. The participants were asked about access to care, antenatal care experience, person-centered approach, and general satisfaction with the facility using a pre-designed questionnaire. These themes were mapped onto the universal Respectful Maternity Care charter. Descriptive statistics were used to summarize the findings in each of these themes. Multivariable logistic regression techniques to determine the relationship between the dependent and independent variables.

**Results:**

There were 904 women who agreed to participate in this study during January to December 2021. Majority of the women (94%, n=854) were satisfied with the operating hours and cleanliness. More than 90% of the women reported positive experiences regarding privacy, respectful treatment by midwives, and non-discriminatory care. However, 40% (n=362) of the women reported not receiving adequate information and informed consent before a medical procedure, while 65% (n=587) reported poor counseling for birth preparedness. Maternal age, women’s occupation, women’s education, and parity were found to be significantly associated with respect provided, satisfaction with counseling and the consent process.

**Conclusion:**

This study reported satisfaction of pregnant women with the facility’s ambiance, respect, and care; however, poor communication skills regarding consent and antenatal counseling were reported. The findings suggest the need for more efficient strategies, such as regular respectful maternity care and technical training to strengthen midwife-patient interactions and enhance overall satisfaction, thus improving maternal and newborn outcomes.

## Introduction

Maternal morbidity and mortality rates remain unacceptably high in low- and middle-income countries (LMICs).^1^ According to the most recent Pakistan Maternal Mortality Survey (PMMS), the maternal mortality ratio (MMR) in Pakistan was 186 deaths per 100,000 live births, with Sindh having the second highest MMR after Baluchistan.^2^ Although Pakistan’s MMR has decreased over the years, efforts must be doubled if we are to meet the Sustainable Development Goal (SDG) targets by 2030.^3^

Antenatal care (ANC) plays a critical role in preventing maternal morbidity and mortality.^4^ It offers a range of services that include administering preventive measures such as immunizations and educating pregnant women on healthy behaviors during pregnancy and identifying and managing potential complications. Despite its benefits, the perception and experiences of pregnant women significantly influence the utilization of ANC services.^4^ Low satisfaction rates with the quality of care offered at the ANC facilities can discourage women from attending ANC visits, leading to missed opportunities for preventive measures and timely management of high risk pregnancies.^5^ Negative perceptions and experiences are often attributed to poor interactions with health professionals, inadequate provision of health information, delayed care, limited involvement in decision-making, and suboptimal conditions of the health facilities, including lack of privacy and cleanliness.^6^ These factors have been associated with negative ANC experiences, leading to ANC dropouts during pregnancy.^7^,^8^

To tackle the high burden of poor maternal outcomes in Pakistan along with the scarcity of a trained healthcare force,^9^ the government has implemented various programs, including the Lady Health Workers and Community Midwives (CMW) programs, to address the shortage of skilled health workers.^10^ These models are widely recognized as a progressive, sustainable, and cost-effective approach to improving maternal and newborn outcomes. However, these seem to be more focused on clinical knowledge rather than technical and soft skills.^11^,^12^ This is also evident as a gap in the literature regarding pregnant women’s experiences with midwifery-led ANC services in Pakistan, which would be an important indicator of uptake of these services.

One of the frameworks that can be used to understand this experience is the Respectful Maternity Care (RMC) charter. This charter supports the rights of pregnant women and promotes dignified and respectful care during pregnancy.^13^ Implementation of such practices could improve the effectiveness of midwifery-led services in reducing maternal and neonatal mortality and morbidity in underserved areas. Thus, this study aimed to understand pregnant women’s experience with midwifery-led antenatal care services using the Respectful Maternity Care charter in primary health centers in Karachi, Pakistan.

## Materials and Methods

### Study Design and Setting

This cross-sectional study was conducted from January to December 2021 at two primary health clinics (PHCs) located in peri-urban communities of Karachi, Pakistan. These communities (ie, Ibrahim Hyderi and Rehri Goth) are homogenous low-income communities situated in Bin Qasim town of the coastal region of Karachi. These sites are part of the Pregnancy Risk Stratification: Innovation and Measurement Alliance (PRiSMA) study, which is coordinated by George Washington University, whose primary aim is to create a harmonized data set to improve global understanding of key risk factors, vulnerabilities, and morbidity and mortality outcomes during the pregnancy and postpartum period. Each study site has four midwives with a diploma in midwifery who provide ANC services to 20–25 pregnant women per day.

### Population & Eligibility Criteria

Eligibility criteria for PRiSMA study included pregnant women living within the study catchment area, at least 15 years old, with an intrauterine pregnancy verified via ultrasound at less than 20 weeks gestation and provided informed consent. All women enrolled in the PRiSMA study and in their third trimester during the defined study period were considered eligible to participate.

### Sample Size Determination and Sampling Procedure

There was no predefined sample size for the study. Instead, data was collected from all third-trimester women who visited PHC between January and December 2021 and agreed to participate in the study.

### Data Collection, Procedures, and Data Quality Control

As part of the secondary objective of PRiSMA, the women’s experience regarding healthcare accessibility, antenatal care, ANC experience, and overall satisfaction with the facility was recorded on a Likert scale using a pre-designed questionnaire on an android tablet. The questionnaire was developed based on the Demographic Health Survey (DHS) Program Service Provision Assessment (SPA) CEI tools, the Kenyan person-centered maternity care scale, and the 2015 Nepal Service Provision Survey (SPA) Client Exit Interview.^14–16^ After obtaining written informed consent in the local language (Urdu) for the interview, an independent study staff member with at least 12 years of education conducted in-person interviews with the study participants.

To ensure the quality of data collected, the data collectors underwent a rigorous two-day training on the questionnaire, data collection procedures, and ethical considerations. Additionally, study field supervisors conducted spot checks on a random sample of 10% completed questionnaires weekly to verify the accuracy of the collected data.

The study was conducted in accordance with the ethical standards laid down in the 1964 Declaration of Helsinki and its later amendments and was approved by the institutional Ethics Review Committee at The Aga Khan University (2021–5920-15,518).

### Statistical Analysis Plan

For analysis, the questions were mapped onto the universal Respectful Maternity Care (RMC) charter (Table 1), which outlines women’s maternity care rights.^13^ Articles 1 and 7–10 were not covered in the study questionnaire, and hence, only articles 2–6 were included in the analysis. Additionally, questions regarding client’s general satisfaction with the PHC facilities were included as a separate theme, namely “General Facility Satisfaction”.

**Table 1:** Articles of the Respectful Maternity Care (RMC) Model with Their Definitions Matched with Questions from the CEI questionnaire^15^

	RMC Articles	Description of the Articles	Related Questions on the Study Tool
1	Freedom from harm or ill-treatment	Everyone has the right to freedom from harm and ill-treatment.	N/A
2	Right to information	Every woman has the right to information, informed consent and refusal, and respect for her choices and preferences, including companionship during maternity care.	Permission for procedures
			Client involvement in decisions
			Discussion on examination, medications, and complications
			Counseling on nutrition, healthy pregnancy, and birth preparedness
3	Privacy and confidentiality	Everyone has the right to privacy and confidentiality	Client’s privacy and confidentiality
4	Dignity and respect	Everyone is their own person from birth and has the right to be treated with dignity and respect.	Respectfulness, thoughtfulness, attentiveness, and approachability
			Blaming, threatening, and filing complaints
5	Equitable care	Everyone has the right to equality, freedom from discrimination, and equitable care.	Any discrimination based on language or ethnicity
6	Right to healthcare	Everyone has the right to healthcare and the highest attainable health level.	Main decision-maker
			Highest attainable care
			Counseling regarding danger signs, neonatal and postnatal care
			Advice and action plan for labor signs and complications
7	Liberty and autonomy	Everyone has the right to liberty, autonomy, self-determination, and freedom from arbitrary detention.	N/A
8	Right to be with their parents or guardians	Every child has the right to be with their parents or guardians	N/A
9	Identity and nationality	Every child has the right to an identity and nationality from birth.	N/A
10	Right to nutrition and clean water	Everyone has the right to adequate nutrition and clean water	N/A

All statistical analyses were conducted using STATA version 14.2. The descriptive statistics for the study variables were reported using mean and standard deviation for continuous variables such as age, and frequency and percentages for categorical variables such as women’s experiences. The Likert scale “yes all the time” and “yes most of the time” were combined to create a single category labelled as “yes”, the other categories were retained as “no” and “do not know”. Initially, all outcome variables on RMC were assessed at a univariate level against age, woman’s education, woman’s occupation, and parity. Variables that had a p-value of less than 0.25 at the univariate level (refer to Table S1) were included in the subsequent multivariable logistic regression analysis. The stepwise backward elimination method was used to build final model. A p-value of ≤ 0.05 was considered statistically significant.

## Results

A total of 970 women were approached, out of which 904 pregnant women (93%) consented to participate in the study. The mean age of the participants was 27.3 ± 6.2 years, of which 75% (n=673) were multiparous. The baseline characteristics of the study participants are described in Table 2. The study’s results are presented in accordance with the RMC’s themes described in Table 1, along with findings of the client’s general satisfaction with the PHC facilities.

**Table 2:** Baseline Characteristics of Women Enrolled in the Study

Characteristics	Total (n=904)
	n (%)
**Women’s age, years (Mean ± SD)**	27.3 ± 6.23
**Parity**	
Primiparous	231 (25.6)
Multiparous	673 (74.5)
**Women’s Education**	
No formal education	575 (63.6)
Educated	329 (36.4)
**Women’s Employment Status**	
Unemployed	576 (63.7)
Employed	328 (36.3)
**Husband’s Occupation**	
Unemployed	13 (1.4)
Employed	891 (98.6)
**Ethnicity**	
Sindhi	306 (33.9)
Pashtun	223 (24.7)
Urdu speaking	153 (16.9)
Others	222 (24.6)
**Previous Delivery Outcomes^a^**	
Live Birth	613 (91.5)
Still Birth	29 (4.3)
Miscarriage	28 (4.2)
**Previous delivery location^b^**	
Facility	489 (76.2)
Home Birth	153 (23.8)
**Previous mode of delivery^b^**	
Vaginal	533 (83.0)
Cesarean	109 (17.0)
**Current Health Status**	
No comorbidities	837 (92.6)
Women with known comorbidities^c^	67 (7.4)
Chronic Hypertension	38 (4.2)
Renal Disease	20 (2.2)
Hepatitis	10 (1.1)
Diabetes Mellitus	6 (0.7)
Cardiac Disease	4 (0.4)

**Notes**: ^a^3 Not available, and 231 are primiparous. ^b^31 Not available, and 231 are primiparous. ^c^Women may have more than one comorbidities.

### General Facility Satisfaction

In this section, we enquired regarding satisfaction with the ambience and waiting hours at the facility. The most common reasons identified for choosing the PHC facility were encouragement by the field staff (37%, n= 336), familiarity with the midwives (35%, n=320), and the availability of free services (14%, n= 128). Furthermore, the results showed that 97% (n=884) of the participants were satisfied with the operational hours of the facility. Also, 88% (n=797) of the women found the facility to be clean. Half of the respondents (50%, n=456) reported that they had received services at the facility within an hour of waiting. The odds of satisfaction among employed women was three times higher compared to unemployed women (AOR=2.99; 95% CI: 2.08–4.30) (refer to Table 3).

**Table 3:** Multivariate Analysis Showing Factors Associated with Maternal Satisfaction with Antenatal Care in Public Health Centers

	Right to Information			Dignity and Respect	Right to Healthcare
	Satisfaction with Understanding the Need for Examination	Satisfaction with Healthy Pregnancy Counselling	Satisfaction with Nutrition Counselling	Satisfaction with Handling Misconduct or Disrespect	Danger Sign Recall Scores
**Age**	~	~	~	1.04 (1.01–1.07)	1.03 (1.00–1.05)
**Women’s occupation**					
Unemployed	Ref	Ref	Ref	Ref	Ref
Employed	0.75 (0.57–0.99)	2.99 (2.08–4.30)	0.56 (0.43–0.75)	0.17 (0.11–0.28)	0.70 (0.50–0.97)
**Women’s Education**					
Illiterate	Ref	~	Ref	~	~
Literate	1.69 (1.29–2.23)		1.59 (1.21–2.09)		
**Parity**					
Multi Parous	~	Ref	~	~	~
Primary Parous		1.46 (1.01–2.11)			

### Article 2 - Right to Information

**Figure 1:**
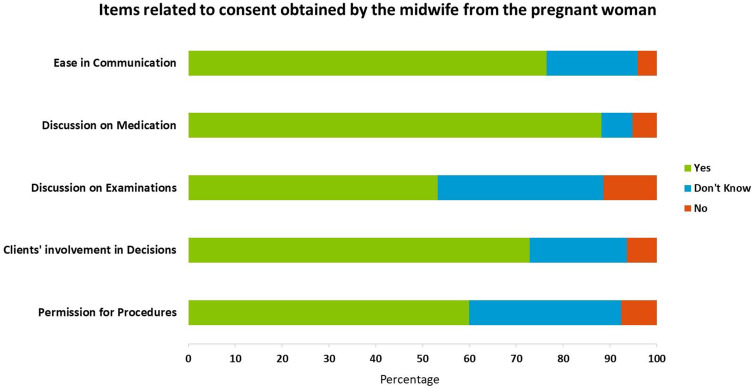
Items related to consent obtained by the midwife from the pregnant woman.

Pregnant women were asked regarding information provided related to current examination. Sixty percent (n=542) of women reported that midwives took permission before performing any exams, and Fifty three percent (n=481) reported understanding the need for those examinations when explained by the midwife (refer to Figure 1). Furthermore, pregnant women also reported high satisfaction with sessions related to a healthy pregnancy (75%, n=677). However, only 43% of the women expressed satisfaction with nutritional counseling (n=395) and 13% with counseling on birth preparedness (n=119).

Study participants who were literate were more likely to report satisfaction with understanding the need for examinations and nutritional counselling than women with no formal education (OR = 1.69; 95% CI: 1.29–2.23) and (OR = 1.59; 95% CI: 1.21–2.09), respectively). Employed women reported lower satisfaction with nutritional counseling (OR=0.56; 95% CI: 0.43–0.75) and with the process of obtaining permission before conducting examinations and procedures (OR=0.17; 95% CI: 0.11–0.28) compared to unemployed women. Additionally, women with a high parity were more likely to recall healthy pregnancy counselling than women who were pregnant for the first time (OR = 1.46; 95% CI: 1.01–2.11).

### Article 3 - Privacy and Confidentiality

The majority of pregnant women (99%, n=892) reported that their privacy was ensured by providing a cover or screen during the examination.

### Article 4 - Dignity and Respect

Pregnant women reported high satisfaction with respectfulness (99%, n=899), friendliness (99%, n=898), and addressing women by their name (100%, n=900) by the midwives at the health facility. Even though majority of the women felt respected when asked if they would take any action against the care provider in case they felt wronged or disrespected, 41% (n=374) reported that would prefer not to take any action. Employed women were less satisfied as compared to unemployed women regarding respect provided (OR = 0.17, 95% CI: 0.11–0.28) and increasing age was associated with a higher likelihood of feeling respected (OR = 1.04; 95% CI: 1.01–1.07) than their counterparts.

### Article 5 - Equitable Care

Majority of pregnant women felt that staff spoke to them in a language they understood and did not feel any discrimination based on ethnicity or educational status (99%, n=897 and 97%, n=880), respectively.

### Article 6 - Right to Healthcare

**Figure 2:**
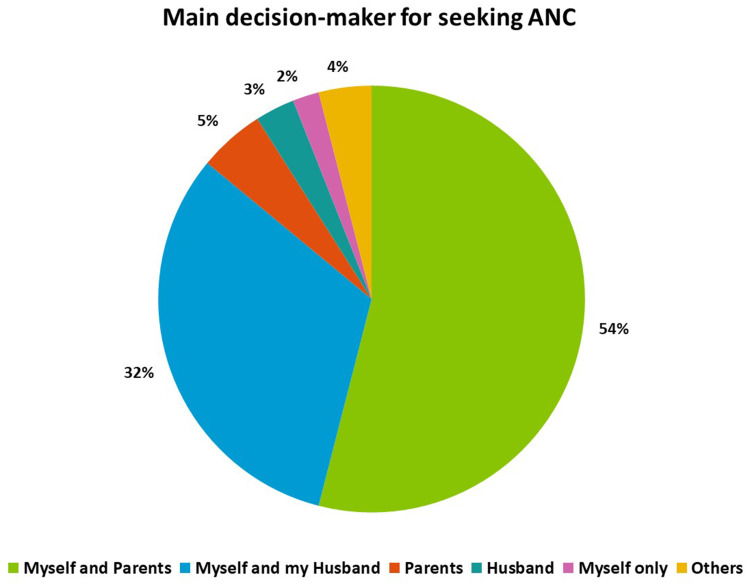
Main decision-makers for seeking ANC.

The distribution regarding decision-making to seek ANC at the health facility has been shown in Figure 2. Approximately 34% (n=309) reported that they decided to seek care either with their husbands or independently. To assess whether pregnant women had access to their right to healthcare and the highest attainable level of health, they were evaluated based on their ability to recall counseling regarding complications during pregnancy, labor and delivery, and newborn counseling provided by the midwife. Fifty-nine percent of women (n=479) were able to recall at least four danger signs out of eight. Additionally, fifty-nine percent of women (n=536) were able to recall all three labor signs, whereas only 39% (n=351) women were able to recall any neonatal danger signs. In the event of an emergency, 50% of women (n=454) reported they would call a midwife, while the remaining (48%, n=430) said they would attempt to visit the health center or referral hospital.

## Discussion

This study reported pregnant women’s satisfaction with midwifery-led services at a PHC in a peri-urban community in Pakistan. Women reported high satisfaction with respect to general facility conditions, provider attitude and service, and availability of timely care at the PHC. However, findings suggested that pregnant women’s right to information, informed consent, and preferences needed significant improvement with respect to the provider-patient encounter.

Midwives are potential solutions to skilled-care provider shortages and have been shown to significantly prevent and lower morbidity and mortality in mothers and newborns.^11^ This study aimed to understand the experience of pregnant women on RMC attributes in our health facility. The RMC Charter is a global advocacy tool aimed at strengthening the rights of mothers and their newborns in relation to the maternity care offered at health facilities.^13^ It served as a framework for mapping the study tool to understand the experience of pregnant women with RMC attributes in our health facility. Most studies have assessed the impact of the RMC charter as a model for promoting good practices during intrapartum and postpartum care.^13^,^17^ In contrast, the current study focused on assessing the gaps during the ANC period.

The facility’s physical infrastructure, regarding waiting areas and overall cleanliness, is known to impact women’s ANC experience significantly.^18^ Even though there were some concerns with waiting time of > 30 minutes, which have shown to have a negative relationship with women’s satisfaction and poor compliance to ANC visits,^19^ the current study reported high satisfaction with the facility’s general services and cleanliness.

In more traditional cultures such as ours, mothers-in-law or elders have the authority to make pregnancy-related decisions, particularly for young women.^20^,^21^ The current study saw a similar trend where women’s ability to seek ANC services and make independent decisions was much lower (2.5%) and was instead influenced by their increasing age and parity. This poor decision making also extends during the patient provider encounter where patients often feel unable to provide fully informed consent for procedures and examinations due to the lack of adequate debriefing by health providers and insufficient opportunities for involvement in decision-making.^22^,^23^ The lack of this decision-making ability also violates the patient’s rights specified by the World Health Organization.^24^ Eight percent of women in the current study reported that midwives performed exams or procedures without obtaining consent. However, 99% of the women reported that their privacy and confidentiality were maintained throughout the ANC encounters. Notably, the lack of literature on patients’ experiences during antenatal visits in LMIC further distinguishes this study.^25^

The current study reported that, while most women had better recall of pregnancy danger signs and labor signs, their retention of information regarding healthy pregnancy, nutritional counseling, and newborn care was comparatively poor. This is consistent with findings by Assaf et al, who suggested that the recall patterns could be due to lack of attention by pregnant women during information provision or midwives imparting excessive information in a short period, thus overwhelming the women.^26^ Poor literacy status of pregnant women (as seen in the current study) may be a contributing factor to the knowledge gaps identified.^27^ At the provider’s end, midwives in such settings may need more time to perform routine check-ups as well as offer counseling to every woman at each visit.^28^ Further, in the study context, postnatal care is mainly provided at home through community health workers. As a result, the midwives may not prioritize counselling on this aspect, as it falls outside of their direct responsibility. Regardless, the charter requires providers to switch from a disease-focus to a patient-centered approach and improve their interpersonal skills to adhere to the RMC standards.^13^ Findings from one study highlight the potential for tailored interventions such as the use of visual aids, culturally appropriate materials in local languages, and group education sessions led by community-based volunteers along with efficient governmental policies in improving health knowledge among women with limited literacy skills.^29^

Due to the limited literature available in this area, the current study adds to the evidence of adherence to the RMC charter in a midwifery-led facility in a low-resource setting. These findings may have implications beyond Pakistan, including increased awareness and advocacy for midwifery practice in countries with similar maternal and child health challenges. The study is limited in terms of its sample, as it was conducted in a single region of Pakistan, which may limit its generalizability to the wider population. Even though we tried to maintain privacy, there may be potential respondent bias. Further, a solely quantitative approach may not comprehensively capture the women’s perception of care. Despite these limitations, the study provides valuable insights and highlights areas for further research and improvement.

## Conclusion

The study findings provide a nuanced perspective on the significance of understanding the pregnant women’s experience in midwifery-led care in an LMIC. This study has highlighted opportunities to improve midwives’ compliance with RMC attributes, thereby improving the patient-provider encounter. RMC training, in addition to technical training, would assist midwives in improving their knowledge of routine maternity care procedures and adopting a patient-centered approach, potentially leading to significant improvements in practice and maternal and newborn health outcomes.

## Abbreviations

ANC, Antenatal care; PHC, Primary Health Centre; RMC, Respectful Maternity Care; LMICs, Lower-Middle-Income-Countries; PMMS, Pakistan Maternal Mortality Survey; CMW, Community Midwife; MMR, Maternal Mortality Ratio; SDG, Sustainable Development Goals; CEI, Client Exit Interview; DHS, Demographic Health Survey; SPA, Service Provision Assessment; BMGF, Bill & Melinda Gates Foundation; PRiSMA, Pregnancy Risk Stratification Innovation and Measurement Alliance.

## Data Sharing Statement

Researchers may request access to anonymized participant-level data from the corresponding author.
